# The Evolving Role of Ultrasound Guided Percutaneous Laser Ablation in Elderly Unresectable Breast Cancer Patients: A Feasibility Pilot Study

**DOI:** 10.1155/2018/9141746

**Published:** 2018-06-11

**Authors:** Jacopo Nori, Maninderpal Kaur Gill, Icro Meattini, Camilla Delli Paoli, Dalmar Abdulcadir, Ermanno Vanzi, Cecilia Boeri, Silvia Gabbrielli, Elisabetta Giannotti, Francesco Lucci, Vania Vezzosi, Diego De Benedetto, Giulia Bicchierai, Simonetta Bianchi, Luis Sanchez, Lorenzo Orzalesi, Guido Carmelo, Vittorio Miele, Lorenzo Livi, Donato Casella

**Affiliations:** ^1^Diagnostic Unit, Azienda Ospedaliero-Universitaria Careggi, Florence, Italy; ^2^Department of Biomedical Imaging, Kuala Lumpur General Hospital, Kuala Lumpur, Malaysia; ^3^Radiation Oncology Unit, Azienda Ospedaliero-Universitaria Careggi, University of Florence, Florence, Italy; ^4^Division of Pathological Anatomy, University of Florence, Florence, Italy; ^5^Breast Surgery Unit, Azienda Ospedaliero-Universitaria Careggi, University of Florence, Florence, Italy; ^6^Anesthesia Unit, Azienda Ospedaliero-Universitaria Careggi, Florence, Italy

## Abstract

**Background and Objectives:**

Breast-conserving surgery represents the standard of care for the treatment of small breast cancers. However, there is a population of patients who cannot undergo the standard surgical procedures due to several reasons such as age, performance status, or comorbidity. Our aim was to investigate the feasibility and safety of percutaneous US-guided laser ablation for unresectable unifocal breast cancer (BC).

**Methods:**

Between December 2012 and March 2017, 12 consecutive patients underwent percutaneous US-guided laser ablation as radical treatment of primary inoperable unifocal BC.

**Results:**

At median follow-up of 28.5 months (range 6-51), no residual disease or progression occurred; the overall success rate for complete tumor ablation was therefore 100%. No significant operative side effects were observed, with only 2 (13.3%) experiencing slight to mild pain during the procedure, and all patients complained of a mild dull aching pain in the first week after procedure.

**Conclusions:**

Laser ablation promises to be a safe and feasible approach in those patients who are not eligible to the standard surgical approach. However, longer follow-up results and larger studies are strongly needed.

## 1. Introduction

Breast cancer (BC) is the most frequently occurring cancer in women (28.8% of all cancer diagnosis) and the second most common in the world, with an estimated lifetime risk of 1/10 women [1].

Despite the steady increase of the number of BC newly diagnosed worldwide, its mortality has shown a slight decrease in western countries. This might be due to widespread screening programs, resulting in an increased diagnosis of small tumors, and it might be secondary to better therapeutic strategies [2, 3]. Over 40% of women with newly diagnosed BC are aged 65 years or older, and the median age at diagnosis is around 60 years [4].

Although breast-conserving surgery (BCS) represents the treatment of choice for small BC [5], elderly women often receive less than this standard therapy. In a large study, which involved over 120,000 women, decreased surgical rates were associated with higher age due to several reasons such as performance status or comorbidity [4, 6, 7].

Therefore, in the last decade, in parallel to the increasing age of patients with a diagnosis of breast cancer, there has been a progressive demand for minimally invasive treatments specifically aimed at BC [8–10]. These treatments are not substitutes for breast surgery, which is still the primary treatment of choice, but they could represent a radical treatment for the nonoperable group of patients or for those who refuse surgery. The approaches available include cryoablation [11–15], radiofrequency [16, 17], microwave ablation [18], focused ultrasound (US) [19, 20], and laser ablation [21, 22].

The purpose of our single-center retrospective study is to assess the feasibility of ultrasound guided percutaneous laser ablation (LA), as the treatment of small unifocal breast cancer in nonoperable elderly patients and in patients who refuse surgery.

## 2. Methods

We achieved the Internal Review Board (IRB) approval for this trial, performed in a large university referral hospital for breast disease. Written informed consent of patients was required for the inclusion in this study. All procedures were in accordance with the ethical standards of the institutional and/or national research committee and with the 1964 Helsinki Declaration and its later amendments or comparable ethical standards.

The primary objectives of our study are to (i) describe the results of laser ablation in nonoperable patients with unifocal BC and (ii) assess the effectiveness and safety of LA to treat BC.

The secondary objectives would be to (i) evaluate the breast cosmesis, (ii) determine regional and distant breast tumor recurrence rate up to 5 years, and (iii) determine the overall survival rate among the period of the study.

### 2.1. Patient Selection and Period of Study

We retrospectively reviewed 12 breast cancer patients who underwent percutaneous procedures in our Department of Breast Diagnostic Senology Unit of the Careggi Hospital, Italy, between December 2012 and March 2017. The multidisciplinary team of our department carefully selected elderly patients (>75 years), with inoperable breast cancer, due to comorbidities and/or to high anesthetic risks, or who refuse surgery.

The inclusion criteria were as follows:

1. unresectable unifocal BC due to comorbidity (e.g., severe cardiovascular or respiratory comorbidities, age, performance status),
2. tumor size ≤ 20 mm in the greatest diameter,
3. the necessity of lesion being US visible at the time of treatment,
4. tumor located at least 1.0 cm from the chest wall as well as the skin and nipple at USG,
5. a biopsy proving invasive ductal unifocal, mucinous, or tubular carcinoma.

The exclusion criteria were as follows:

1. patients suitable for surgery or radiation approach,
2. patients with multifocal or multicentric tumors,
3. absence of written informed consent.

### 2.2. Procedure Planning

All patients underwent clinical and radiological assessments prior to the laser ablation. Clinical assessment included physical breast examination to exclude skin or nipple involvement.

### 2.3. Breast Mammogram and Ultrasound

Radiological assessment included a bilateral two-projection 2D-3D mammography (MMG) (Selenia® Dimensions®, Hologic®, Bedford, USA) and ultrasonographic (US) examinations were performed using a 10–13 MHz transducer and a US unit (ESAOTE, MyLab 70 XVG, Genoa, Italy).

### 2.4. Ultrasound Guided Biopsy

All lesions were sampled with US-guided core-needle biopsy (CNB) using a 10 cm 14-gauge cutting needle with a 22 mm throw (Precisa™, HS® Hospital Service, Rome, Italy). A mean of 4 samples (range 3-5 samples) was taken in each case to evaluate the histological and biological parameters of the tumor. Specimens underwent a standard histological evaluation.

### 2.5. Laser Ablation Procedure

The procedure needed an ultrasound interventional suite. Procedures were performed by using a commercially available US system with an integrated laser source with a 1064 nm wavelength (EchoLaser; Elesta, Calenzano, Italy).

In each case, procedure needed the presence of an anesthesiologist in the ultrasound interventional room and available venous access. Generally, patients underwent local anesthesia; we used conscious sedation only if indicated.

**Figure 1:**
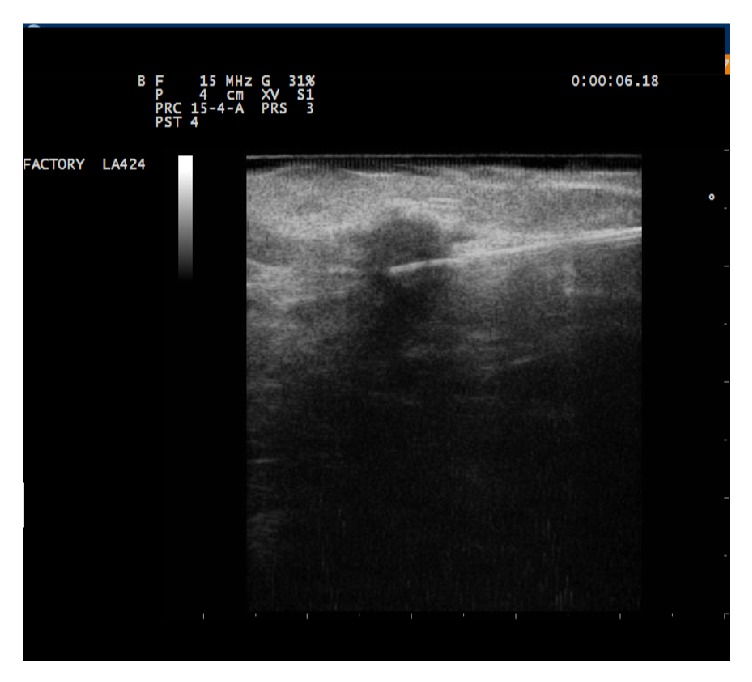
The device LA (introducer-needle and fiber) must be progressively inserted towards the target, choosing the best path to correctly position the tip of the fiber. It is necessary to ensure that the path of the applicator is as parallel as possible to the chest wall. The tip of the device should always be inserted at the center of the lesion and its position must always be controlled with two-plane ultrasound images.

The operator inserts the 21G spinal needle in the most suitable direction to reach the lesion following the shortest possible path. Then the operator introduces and advances a 300 *μ*m flat-tip laser fiber to the tip of the needle. The introducer-needle was designed to expose the fiber tip of 5 mm. The procedure must provide a safe distance of 1.0 cm from the skin and 1.0 cm from the chest wall. The operator must progressively move the device LA (introducer-needle and fiber) towards the target, choosing the best path to correctly position the tip of the fiber. It is necessary to make sure that the path of the applicator is as parallel as possible to the chest wall. The tip of the device must always be in the center of the lesion and its position must always be controlled with two-plane ultrasound images (Figure 1).

According to previous experiences of other authors in other applications of the thin needle laser methodology, each treatment was performed with a fixed power protocol (3W), modifying the lighting time according to the size of the tumor. Depending on the size of the tumor at baseline, the operator performs one or two consecutive illuminations with a “pull-back” technique during the same treatment session. The treatment ends when the gas, formed during the ablation, covers the entire desired area or up to 1800 J for illumination. Each ablation time varies from a minimum of 600 seconds (for tumor size up to 1.0 cm) to a maximum of 1200 seconds (for tumor size between 1.0 and 2.0 cm), in order to maintain the total energy applied between 1800 and 3600 J, respectively [23–26]. Patient vital signs monitoring was continuous during all of the procedure.

In all cases, the laser fiber was active during the retraction of the needle, in order to prevent a possible seeding of tumor cells along the needle tract. Patient monitoring continued up to 2–4 hours, in order to exclude unexpected acute complications, and patient discharge occurred subsequently, during the same day.

### 2.6. Follow-Up of Patients

Clinical follow-up began after 1 week, then at 3 months, and every 6 months until the fifth year. Clinical examination assessed the skin and nipple conditions and it evaluated the clinical size of the treated lesion, if palpable. Radiological follow-up included a weekly US examination from the 1st and 4th week after the ablation procedure. Follow-up also included bilateral mammography and ultrasound after 6 months from the laser procedure and every 12 months thereafter up to 5 years.

The radiologist who performed the LA procedure was the same that performed the ultrasound and mammographic image evaluation. We have observed modifications of treated site after the procedure, and we have considered as suggestive of ablation/recurrence the radiologic aspects observed. Complete ablation corresponded to a well-demarcated area of coagulation zone at the previously ablated site on ultrasound. Definition of recurrences were as follows:

*(1) Local*

- The previously ablated area had an ill-defined margin, and a soft tissue echogenicity within 10 mm of the ablated margin was seen.
- The previously ablated region increased in size.

*(2) Distant*

- New lesion was at >10 mm from the ablated margin.

### 2.7. Complications

Major complications were related to admission to the hospital for therapy, an unplanned increase in the level of care, prolonged hospitalization (more than 3 days), permanent adverse sequelae, or death. Any other complication was considered minor.

We have recorded complications of treatment according to the Society of Interventional Radiology (SIR) guidelines [27, 28].

### 2.8. Survival

Overall survival was defined as the time from the initial laser ablation session until death or the last patient contact. The follow-up for this study ended in March 2017.

## 3. Results

### 3.1. Demographic Analysis of Study Population

Data of 12 patients with invasive breast cancers are summarized in Table 1. Their mean age was 79.2 years (range 75-92). All patients were postmenopausal.

They have completed preliminary diagnostic phase in a mean of 11 days before the laser treatment (range 1-60), and they underwent laser ablation in the US-dedicated room, through local and conscious anesthesia. Mean US-based ablated lesion size was 12.72 mm (range 0.7-20).

Five (41.7%, 5/12) tumors were located in the right breast while seven (58.3%, 7/12) tumors were in the left breast. The pathological diagnoses of the carcinomas were 10 ductal infiltrating (83.3%, 10/12), 1 mucinous (8.3%, 1/12), and 1 tubular (8.3%, 1/12).  Table 2 reported the clinical and US assessment prior to ablation.

### 3.2. Laser Ablation Analysis

The fibers were placed into the center of the lesion of each tumor, and LA was completed according to a planned protocol in all sessions with a technical success rate of 100 %. The overall treatment time ranged from 20 to 35 minutes.

Response to the treatment was evaluated using US after 1 to 4 weeks, with US and mammogram at 6 months, and then annually up to 5 years.

**Figure 2:**
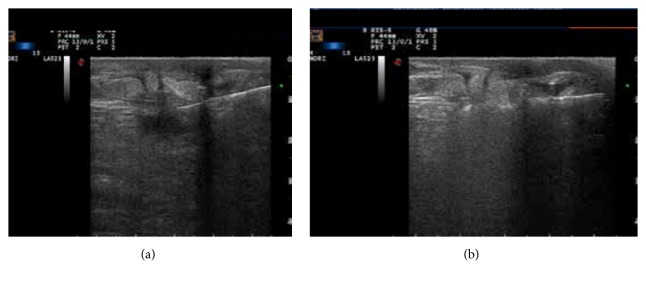
Representative case of successful LA ablation in a patient with invasive ductal unifocal breast carcinoma of 18 mm of max diameter in the upper outer quadrant of the right breast. (a) The US image before treatment shows the hypoechoic lesion with ill-defined margins and the fine needle and the tip of the fiber in the outer third of the tumor mass. (b) The US image at the end of the treatment shows an evident shadow cone due to the presence of gas bubbles that completely cover the ablated area.

**Figure 3:**
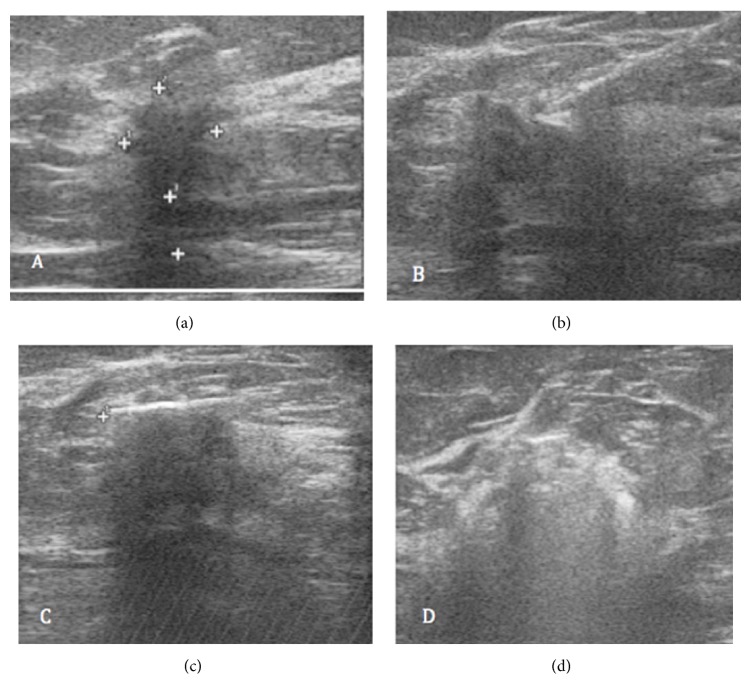
Another example of successful LA ablation in a patient with invasive ductal carcinoma of 15 mm of max diameter in the upper outer quadrant of the right breast. (a) The US image before treatment shows the hypoechoic lesion with blurred margins. (b) The US image shows the laser applicator (21G needle and fiber) which, with a course parallel to the chest wall, reaches the outer edge of the lesion. (c) Finally, the lesion is no longer appreciable, and in the treated area, there is an echogenic line with an evident shadow cone. (d) The US image of the ablated area in the first hours after treatment appears in the form of a heterogeneous predominantly hyperechoic zone (gas bubbles) with blurred margins.

**Figure 4:**
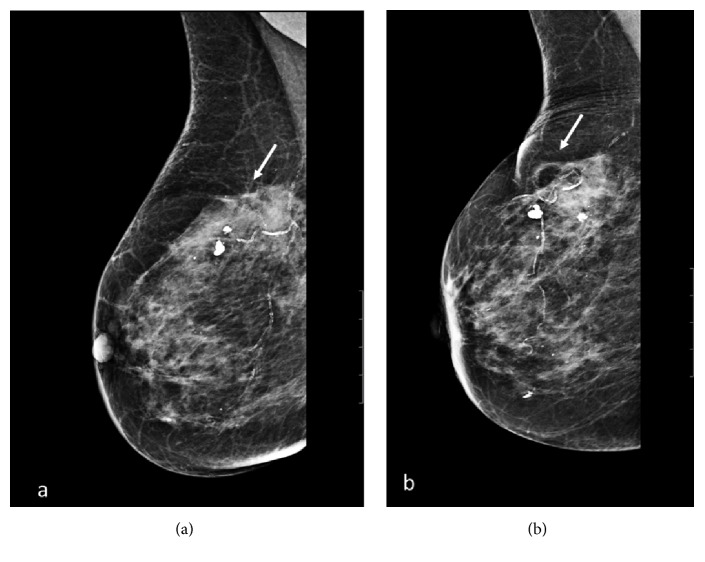
Sequential mammograms showing the cystic oil formation by steatonecrosis over a period of 24 months. (a) Before LA (white arrow) and (b) 24 month after a single laser treatment (white arrow).

At the site of ablation, all lesions showed a well-demarcated cystic lesion, visible at the 6-month US and compatible with coagulative necrosis. The overall success rate for complete tumor ablation (CTA) was 100% (Figures 2-3). At mammography, the necrotic lesion appeared as a typical area of steatonecrosis (Figure 4).

Follow-up lasted a mean time of 28.5 months (range 6-51). None of the patients demonstrated evidence of local or distant recurrence during follow-up. No breast cancer related deaths occurred in any of the patients.

### 3.3. Complications

During the procedure, 2 (16.6%, 2/12) patients complained of mild pain and required conscious sedation. The remaining patients well tolerated the procedure with local anesthesia. However, all the patients experienced a minimal aching sensation at the ablation site during the first week after the procedure (SIR class A) [27, 28]. There were no complications of skin burns in the posttreatment clinical follow-up. The overall treatment time ranged from 20 to 35 minutes. Patients did not need hospitalization; they underwent procedure and they discharged the same day.

None of the patients sustained any systemic adverse effects and there was no evidence of postablation hematoma, infection, or skin burns.

## 4. Discussion

The main advantages of minimally percutaneous therapies included the non-invasiveness, the good cosmesis, the lower painfulness, the short recovery time, and the possibility of a daycare procedure. These factors reduce the cost of hospital stay and they potentially lower risks of morbidity and mortality, in this group of elderly patients.

Our study showed that percutaneous laser ablation is a feasible and effective option for selected unresectable BC patients. We managed to obtain complete ablation in patients with small lesions (T1 lesions ≤ 20 mm). We excluded invasive lobular carcinoma and ductal carcinoma in situ, due to the unfavorable results with other minimally invasive techniques, as shown by previous reports [22].

In line with the above-mentioned studies, we chose US to guide the treatment, since it was effective in showing the real-time correct positioning of the needle at the center of the lesion and the change in echogenicity during treatment. In all cases, the increased echogenicity had an effect of partial hiding of the treatment area (fog-effect). This is due to high temperatures close to the fiber tip when it reaches over 100°C and the secondary formation of air bubbles [22]. The choice of the amount of energy administered to ablate the treated lesions accorded with previous reports [23–26].

The choice of lesions up to 20 mm in the greatest diameter is due to the waited association between the success rate of laser ablation and tumor size. The fine needle approach offered the maximum flexibility and it allowed a tailored approach to the characteristics and location of the tumor. Increased experience of the radiologist could lead to the ablation of larger lesions. None of our patients demonstrated a radiological disease progression.

A breast MRI would have been an ideal contrasted baseline examination in addition to the mammogram and ultrasound, since it represents one of the most sensitive techniques to assess the real extention of the lesion [29, 30]. However, in our group of patients, considering the age factor and associated comorbidities of renal failure and pacemakers in situ, we could not perform an MRI as a baseline examination to all patients. This can represent a limitation of our study.

Other limitation is related to the type of evaluation after treatment, depending only on the imaging, without histological confirmation of the response to the treatment. A future prospective study is going to need a longer follow-up and an evaluation of efficacy, proved through a biopsy after treatment.

Moreover, in other case reports about small lesions (up to 15 mm), authors have reported response rates to ablative technique through mammography and US [21].

Less than 15% of our patients showed complications as mild pain during procedure, while all the patients complained of a mild dull aching pain after procedure. Long-term cosmetic result was also satisfactory. Among the limitations of the study, there is the small number of patients, highly selected in the group of elderly patients affected by unifocal tumors who are nonsurgical candidates. However, through our pilot study, we can state that laser ablation can be usefull and feasible in the treatment of single small breast cancers with complete necrosis of the lesion, good cosmetic outcome, and cost effectiveness.

## 5. Conclusion

Laser ablation is a feasible, minimally invasive, and cost-effective alternative for a subset of patients affected by small lesions, who are not eligible to the standard surgical approach, as well as for patients who refuse surgery. However, further larger prospective studies are strongly needed in order to confirm our preliminary results.

## Figures and Tables

**Table 1:** Data of patients and diagnostic findings prior to treatment.

**Characteristic**	**Value**
Median age (range)	79.25 (75-92)

Postmenopausal, % (*n*)	100 (12/12)

Right breast, % (*n*)	41.7%, 5/12

Left breast, % (*n*)	58.3%, 7/12

Median ultrasound tumor size (mm) (range)	12.72 (range 0.7-20)

Histology, % (*n*)	

(i) Ductal carcinoma	83.3% (10/12)

(ii) Mucinous carcinoma	8.3% (1/12)

(iii) Tubular carcinoma	8.3% (1/12)

**Table 2:** Patients characteristics.

**Patient Number**	**Reason for non-operability**	**Age (Years)**	**USG tumor size (Pre-biopsy) (mm)**
01	Hypertension (HTN) and diabetes mellitus (DM)	80	15 mm

02	Congestive heart failure with DM	88	15 mm

03	CVS co-morbidity and HTN	83	10 mm

04	Ischemic heart disease	84	15 mm

05	DM with end stage renal disease	87	12 mm

06	Cardiomyopathy	86	10 mm

07	Patient refused operation	90	7 mm

08	CVS co-morbidity and pacemaker	85	15 mm

09	Patient refused operation	75	11 mm

10	Age factor with diabetes and hypertension	92	12 mm

11	Parkinson's	90	20 mm

12	Congestive heart failure	88	15 mm
